# Sociodemographic and HIV-Related Characteristics Associated with Mental Health Diagnoses Among People Living with HIV

**DOI:** 10.1007/s10461-025-05015-z

**Published:** 2026-01-09

**Authors:** Monique J. Brown, Jiayang Xiao, Xueying Yang, Banky Olatosi, Sharon Weissman, Xiaoming Li, Jiajia Zhang

**Affiliations:** 1 Department of Epidemiology and Biostatistics, Arnold School of Public Health, University of South Carolina, 915 Greene Street Discovery I, 435C, Columbia, 29208 SC USA; 2 Arnold School of Public Health, South Carolina SmartState Center for Healthcare Quality, University of South Carolina, Columbia, SC USA; 3 Rural and Minority Health Research Center, Arnold School of Public Health, University of South Carolina, Columbia, SC USA; 4 Office for the Study on Aging, Arnold School of Public Health, University of South Carolina, Columbia, SC USA; 5 Centre for Health Systems Research & Development, University of the Free State, Bloemfontein, South Africa; 6 Department of Health Services Policy and Management, Arnold School of Public Health, University of South Carolina, Columbia, SC USA; 7 Department of Internal Medicine, School of Medicine, University of South Carolina, Columbia, SC USA; 8 Department of Health Promotion, Education, and Behavior, Arnold School of Public Health, University of South Carolina, Columbia, SC USA

**Keywords:** Depression, Anxiety, HIV, Risk factors

## Abstract

Mental health diagnoses have been linked to poor HIV treatment outcomes and poorer quality of life among people living with HIV (PLWH). Therefore, this study aimed to investigate the association between sociodemographic and HIV-related characteristics, and common and serious mental health disorders among PLWH in South Carolina (SC). Data were obtained from the integrated system of statewide electronic health record (EHR) data in SC (2006–2019; *N* = 8,124). Multivariable logistic regression models were used to determine the associations between sociodemographic and HIV-related characteristics, and common mental health disorders and serious mental health disorders. Among the study population, 4% were 60 and older, 22% were female, 17% lived in rural areas, and 10% were virally suppressed. Approximately 19% had common mental health disorders while 7% had serious mental health conditions. Sex, race, population type, alcohol use, viral load and comorbidities were associated with having a common mental health diagnosis and serious mental health diagnosis. Mental health intervention and prevention programs should consider addressing these factors and the specific populations that are greatly impacted by adverse mental health outcomes.

**Supplementary Information:**

The online version contains supplementary material available at 10.1007/s10461-025-05015-z.

## Introduction

Adverse mental health outcomes are common among people living with HIV (PLWH) [1, 2] and rates of mental health problems are higher among PLWH compared to the general population [3]. For example, previous research has shown a higher prevalence of depression among PLWH compared to the general population [4]. Depression prevalence estimates are three to eight times higher among PLWH than the general population ranging from 12% to 67% [5–9]. Anxiety prevalence also ranges from 0.6% to 68.2% depending on the specific population of PLWH [2].

Adverse mental health outcomes may negatively impact HIV treatment outcomes such as retention in care, antiretroviral therapy (ART) adherence, and viral suppression [10]. PLWH may also have HIV-related stress, which has been shown to be positively associated with depression [11]. Anxiety is a risk factor for cognitive impairment and is related to greater suicidal behavior among PLWH [12–14]. Anxiety has also been linked to a higher rate of antiretroviral failure, even after viral suppression has been obtained [15]. Untreated depression and anxiety have also been shown to be associated with a lack of viral suppression among PLWH from rural Florida [16].

Among a US Southeastern population in North Carolina, the prevalence of mental health disorders was 60% with co-occurring mental illness and substance abuse at 23% [17]. In addition, among PLWH in a large academic medical center in the Southeastern US, close to four in ten (39%) had a mood/anxiety diagnosis and more than one in five (21%) had a substance use disorder [18]. Having a psychiatric disorder was also shown to be associated with a slower rate of attaining viral suppression among PLWH initiating ART in an academic mental center [15].

However, more recent research assessing factors associated with mental health diagnoses among PLWH in the Southeastern US is needed. Therefore, it is crucial to determine factors associated with mental health diagnoses among PLWH in this geographic location. Therefore, the aim of the study was to examine the association between sociodemographic and HIV-related characteristics, and common mental health disorders and serious mental health disorders among PLWH in South Carolina (SC).

## Methods

### Data Source and Study Population

Data were extracted from the integrated system of statewide electronic health record data in SC to form a retrospective cohort of PLWH. Since 1986, the SC Department of Public Health (SC DPH) has implemented an enhanced HIV/AIDS reporting system (eHARS) that collects confidential name-based reports of HIV/AIDS. The de-identified EHR data from SC DPH’s eHARS, along with claim data from all payers, were linked by the South Carolina Revenue and Fiscal Affairs Office (SC RFA).

In this study, we extracted data from all adult PLWH (18 years or older at the time of HIV diagnosis) who: (1) were diagnosed with HIV in SC between January 1 st, 2006 and December 31 st, 2019, (2) had at least one year of follow-up since their HIV diagnosis, (3) had at least two HIV viral loads (VL) and/or at least two CD4 tests between the time of HIV diagnosis and the end of study or death, whichever came first, and (4) no mental health diagnosis within one year prior to or at the HIV diagnosis. Overall, 8,124 PLWH were included in this study. The study was approved by the University of South Carolina Institutional Review Board (Pro00068124).

## Measures

### Outcome

Mental health diagnosis was identified based on whether the individual living with HIV received a mental health diagnosis ever after HIV diagnosis during follow-up time. Those patients had no mental health diagnosis within one year prior to or at the HIV diagnosis. These diagnoses encompass conditions such as schizophrenia, personality disorder, bipolar disorder, schizoaffective disorders, depression, anxiety, obsessive compulsive disorder, dementia, and persistent mood disorder, with diagnoses referenced through ICD-9/10 codes (Supplemental Table 1). Anxiety or depression were classified as common mental health diagnoses, while any other conditions were categorized as serious mental health diagnoses. These two groups were examined separately.

## HIV-Related Characteristics

The baseline VL and CD4 count measures were determined based on the first measurements on or after the initial HIV diagnosis. The VL measurement was divided into four groups: ≤200 copies/mL; >200 and ≤ 10,000 copies/mL; >10,000 and ≤ 100,000 copies/mL; and ≥ 100,000 copies/mL. The CD4 measurements were categorized into four groups: <200 cells/mm3; 200 to 350 cells/mm3; 350 to 500 cells/mm3; and ≥ 500 cells/mm3. Age at the time of HIV diagnosis, race, and population type were also included.

## Potential Confounders

Additional factors that were considered as possible covariates included sex, and residential area (urban or rural). As for baseline substance use, the study referenced ICD-9/10 codes to categorize and consider baseline alcohol use, amphetamine, antidepressant, cannabis, cocaine use, opioid use, sedative hypnotic or anxiolytic, tobacco use, other psychoactive substance and other stimulant (within one year prior to or at the HIV diagnosis). The analysis also involved identifying and including the number of baseline comorbidities, such as hypothyroidism, hypertension, arthritis, chronic obstructive pulmonary disease (COPD), cardiovascular disease, renal disease, diabetes mellitus, obesity, cerebrovascular disease, dyslipidemia, Hepatitis C, cancer, and Hepatitis B, which were referenced by ICD-9/10 codes.

### Statistical Analysis

We first described the distribution of HIV-related factors and other factors among PLWH who had mental health diagnosis or not during the follow-up period. This analysis was performed using analysis of variance (ANOVA), and Pearson’s Chi-squared test or Fisher’s exact test. Crude and multivariable logistic regression models were used in overall and subgroup analyses to evaluate the association between sociodemographic and HIV-related characteristics, and common mental health diagnosis and serious mental health diagnosis. The Wald test was used to calculate local p-values in the model. As a preliminary analysis, all possible two-way interactions among covariates were tested in the adjusted logistic regression models. All analyses were conducted using R version 4.1.2, and a two-sided p-value of 0.05 was used for statistical significance.

## Results

Table 1 shows the distribution of sociodemographic, substance use, and HIV-related characteristics among the study population and by common mental health diagnosis status. Approximately 19% of the study population had a common mental health diagnosis (depression or anxiety). There were statistically significant differences in age group (*p* = 0.009), sex (*p* < 0.001), race/ethnicity (*p* < 0.001), population type (*p* < 0.001), alcohol use (*p* < 0.001) and substance use (p-value varied depending on substance), VL (*p* = 0.009), and comorbidities (*p* < 0.001) by common mental health diagnosis status. Individuals with a mental health diagnosis tended to be older (aged 40–59), female, White, heterosexual, men who have sex with men/injection drug use (MSM/IDU), use substances at baseline, have a higher baseline VL, and have at least one comorbidity. Table 2 shows the distribution of sociodemographic, substance use, and HIV-related characteristics among the study population and by serious mental health diagnosis status. Approximately 7% of the study population had a serious mental health diagnosis.

**Table 1:** Distribution of sociodemographic, substance use and clinical characteristics by common mental health diagnosis ever after HIV diagnosis

Characteristic	Overall, *N* = 8,124*1*	No, *N* = 6,610*1*	Yes, *N* = 1,514*1*	*p*-value2
Age				**0.009**
18–24	1,939 (23.9%)	1,619 (24.5%)	320 (21.1%)	
25–39	3,256 (40.1%)	2,648 (40.1%)	608 (40.2%)	
40–59	2,575 (31.7%)	2,050 (31.0%)	525 (34.7%)	
≥60	354 (4.4%)	293 (4.4%)	61 (4.0%)	
Sex				**< 0.001**
Male	6,328 (77.9%)	5,343 (80.8%)	985 (65.1%)	
Female	1,796 (22.1%)	1,267 (19.2%)	529 (34.9%)	
Race/Ethnicity				**< 0.001**
White	1,716 (21.1%)	1,257 (19.0%)	459 (30.3%)	
Black	5,750 (70.8%)	4,771 (72.2%)	979 (64.7%)	
Hispanic	442 (5.4%)	399 (6.0%)	43 (2.8%)	
Others	216 (2.7%)	183 (2.8%)	33 (2.2%)	
Population				**< 0.001**
Heterosexual	1,492 (18.4%)	1,126 (17.0%)	366 (24.2%)	
MSM/IDU	383 (4.7%)	267 (4.0%)	116 (7.7%)	
MSM	4,420 (54.4%)	3,719 (56.3%)	701 (46.3%)	
Others	1,829 (22.5%)	1,498 (22.7%)	331 (21.9%)	
Region				0.222
Urban	6,772 (83.4%)	5,526 (83.6%)	1,246 (82.3%)	
Rural	1,352 (16.6%)	1,084 (16.4%)	268 (17.7%)	
Baseline alcohol use				**< 0.001**
No	6,529 (80.4%)	5,429 (82.1%)	1,100 (72.7%)	
Yes	1,595 (19.6%)	1,181 (17.9%)	414 (27.3%)	
Baseline amphetamine use				**0.006**
No	8,104 (99.8%)	6,599 (99.8%)	1,505 (99.4%)	
Yes	20 (0.2%)	11 (0.2%)	9 (0.6%)	
Baseline antidepressant use				0.621
No	8,117 (99.9%)	6,605 (99.9%)	1,512 (99.9%)	
Yes	7 (0.1%)	5 (0.1%)	2 (0.1%)	
Baseline cannabis use				**0.001**
No	8,006 (98.5%)	6,528 (98.8%)	1,478 (97.6%)	
Yes	118 (1.5%)	82 (1.2%)	36 (2.4%)	
Baseline cocaine use				**< 0.001**
No	7,998 (98.4%)	6,528 (98.8%)	1,470 (97.1%)	
Yes	126 (1.6%)	82 (1.2%)	44 (2.9%)	
Baseline opioid use				**< 0.001**
No	8,108 (99.8%)	6,603 (99.9%)	1,505 (99.4%)	
Yes	16 (0.2%)	7 (0.1%)	9 (0.6%)	
Baseline other psychoactive substance use				**< 0.001**
No	8,076 (99.4%)	6,581 (99.6%)	1,495 (98.7%)	
Yes	48 (0.6%)	29 (0.4%)	19 (1.3%)	
Baseline other stimulant use				0.069
No	8,115 (99.9%)	6,605 (99.9%)	1,510 (99.7%)	
Yes	9 (0.1%)	5 (0.1%)	4 (0.3%)	
Baseline sedative hypnotic or anxiolytic use				0.338
No	8,122 (99.9%)	6,609 (99.9%)	1,513 (99.9%)	
Yes	2 (0.1%)	1 (0.1%)	1 (0.1%)	
Baseline tobacco use				**< 0.001**
No	6,545 (80.6%)	5,419 (82.0%)	1,126 (74.4%)	
Yes	1,579 (19.4%)	1,191 (18.0%)	388 (25.6%)	
Baseline VL				**0.009**
≤200	797 (9.8%)	680 (10.3%)	117 (7.7%)	
>200–10000	1,884 (23.2%)	1,517 (23.0%)	367 (24.2%)	
>10,000	5,443 (67.0%)	4,413 (66.8%)	1,030 (68.0%)	
Baseline CD4				0.468
<200	2,340 (28.8%)	1,899 (28.7%)	441 (29.1%)	
200–500	3,457 (42.6%)	2,833 (42.9%)	624 (41.2%)	
>500	2,327 (28.6%)	1,878 (28.4%)	449 (29.7%)	
Number of comorbidities				**< 0.001**
0	6,561 (80.8%)	5,457 (82.6%)	1,104 (72.9%)	
≥1	1,563 (19.2%)	1,153 (17.4%)	410 (27.1%)	

*1* n (%)

*2* Pearson’s Chi-squared test; Fisher’s exact test

Bolded p values are statistically significant at *p* < 0.05

**Table 2:** Distribution of sociodemographic, substance use and clinical characteristics by serious mental health diagnosis ever after HIV diagnosis

Characteristic	Overall, *N* = 8,124*1*	No, *N* = 7,553*1*	Yes, *N* = 571*1*	*p*-value2
Age				**< 0.001**
18–24	1,939 (23.9%)	1,844 (24.4%)	95 (16.6%)	
25–39	3,256 (40.1%)	3,032 (40.1%)	224 (39.2%)	
40–59	2,575 (31.7%)	2,353 (31.2%)	222 (38.9%)	
>60	354 (4.4%)	324 (4.3%)	30 (5.3%)	
Sex				**< 0.001**
Male	6,328 (77.9%)	5,958 (78.9%)	370 (64.8%)	
Female	1,796 (22.1%)	1,595 (21.1%)	201 (35.2%)	
Race/Ethnicity				**< 0.001**
White	1,716 (21.1%)	1,529 (20.2%)	187 (32.7%)	
Black	5,750 (70.8%)	5,388 (71.3%)	362 (63.4%)	
Hispanic	442 (5.4%)	434 (5.7%)	8 (1.4%)	
Others	216 (2.7%)	202 (2.7%)	14 (2.5%)	
Population				**< 0.001**
Heterosexual	1,492 (18.4%)	1,352 (17.9%)	140 (24.5%)	
MSM/IDU	383 (4.7%)	320 (4.2%)	63 (11.0%)	
MSM	4,420 (54.4%)	4,182 (55.4%)	238 (41.7%)	
Others	1,829 (22.5%)	1,699 (22.5%)	130 (22.8%)	
Region				0.558
Urban	6,772 (83.4%)	6,291 (83.3%)	481 (84.2%)	
Rural	1,352 (16.6%)	1,262 (16.7%)	90 (15.8%)	
Baseline alcohol use				**< 0.001**
No	6,529 (80.4%)	6,134 (81.2%)	395 (69.2%)	
Yes	1,595 (19.6%)	1,419 (18.8%)	176 (30.8%)	
Baseline amphetamine use				0.162
No	8,104 (99.8%)	7,536 (99.8%)	568 (99.5%)	
Yes	20 (0.2%)	17 (0.2%)	3 (0.5%)	
Baseline antidepressant use				0.4
No	8,117 (99.9%)	7,547 (99.9%)	570 (99.8%)	
Yes	7 (0.1%)	6 (0.1%)	1 (0.2%)	
Baseline cannabis use				**0.015**
No	8,006 (98.5%)	7,450 (98.6%)	556 (97.4%)	
Yes	118 (1.5%)	103 (1.4%)	15 (2.6%)	
Baseline cocaine use				**< 0.001**
No	7,998 (98.4%)	7,447 (98.6%)	551 (96.5%)	
Yes	126 (1.6%)	106 (1.4%)	20 (3.5%)	
Baseline opioid use				**< 0.001**
No	8,108 (99.8%)	7,543 (99.9%)	565 (98.9%)	
Yes	16 (0.2%)	10 (0.1%)	6 (1.1%)	
Baseline other psychoactive substance use				**0.006**
No	8,076 (99.4%)	7,514 (99.5%)	562 (98.4%)	
Yes	48 (0.6%)	39 (0.5%)	9 (1.6%)	
Baseline other stimulant use				0.128
No	8,115 (99.9%)	7,546 (99.9%)	569 (99.6%)	
Yes	9 (0.1%)	7 (0.1%)	2 (0.4%)	
Baseline sedative hypnotic or anxiolytic use				0.136
No	8,122 (99.9%)	7,552 (99.9%)	570 (99.8%)	
Yes	2 (0.1%)	1 (0.1%)	1 (0.2%)	
Baseline tobacco use				**< 0.001**
No	6,545 (80.6%)	6,134 (81.2%)	411 (72.0%)	
Yes	1,579 (19.4%)	1,419 (18.8%)	160 (28.0%)	
Baseline VL				0.121
≤200	797 (9.8%)	755 (10.0%)	42 (7.4%)	
>200–10000	1,884 (23.2%)	1,746 (23.1%)	138 (24.2%)	
>10,000	5,443 (67.0%)	5,052 (66.9%)	391 (68.5%)	
Baseline CD4				0.287
<200	2,340 (28.8%)	2,167 (28.7%)	173 (30.3%)	
200–500	3,457 (42.6%)	3,232 (42.8%)	225 (39.4%)	
>500	2,327 (28.6%)	2,154 (28.5%)	173 (30.3%)	
Number of comorbidities				**< 0.001**
0	6,561 (80.8%)	6,155 (81.5%)	406 (71.1%)	
≥1	1,563 (19.2%)	1,398 (18.5%)	165 (28.9%)	

*1* n (%)

*2* Pearson’s Chi-squared test; Fisher’s exact test

Bolded p values are statistically significant at *p* < 0.05

Table 3 shows the associations between sociodemographic, substance use, and HIV-related characteristics and common mental health diagnosis among PLWH. Females had twice the odds of having a mental health diagnosis compared to males (adjusted odds ratio (aOR): 2.57; 95% CI: 2.15–3.07). Racially and ethnically minoritized populations all had lower odds of having a common mental health diagnosis compared to White populations (Black: aOR: 0.51; 95% CI: 0.44–0.58; Hispanic: aOR: 0.32; 95% CI: 0.22–0.44; Other: aOR: 0.52; 95% CI: 0.34–0.76). MSM/IDU populations had 50% higher odds of having a common mental health diagnosis compared to heterosexual populations (aOR: 1.50; 95% CI: 1.13–1.99) while Other population type was associated with 19% lower odds of having a common mental health diagnosis (aOR: 0.81; 95% CI: 0.68–0.97). Baseline alcohol use was associated with a higher odds of having a common mental health diagnosis. A higher VL (> 200) was also associated with a higher odds of common mental health diagnosis. Having ≥ 1 comorbidity was associated with higher odds of a common mental health diagnosis compared with having no comorbidities.

**Table 3:** Association between sociodemographic, substance use and clinical characteristics and common mental health diagnosis ever after HIV diagnosis

Characteristic	OR1	95% CI1	*p*-value
Age			
18–24	–	–	
25–39	0.96	0.82, 1.12	0.59
40–59	0.93	0.79, 1.11	0.44
>60	0.73	0.53, 1.01	0.061
Sex			
Male	–	–	
Female	2.57	2.15, 3.07	**< 0.001**
Race/Ethnicity			
White	–	–	
Black	0.51	0.44, 0.58	**< 0.001**
Hispanic	0.32	0.22, 0.44	**< 0.001**
Others	0.52	0.34, 0.76	**0.001**
Population			
Heterosexual	–	–	
MSM/IDU	1.5	1.13, 1.99	**0.005**
MSM	1.05	0.85, 1.29	0.65
Others	0.81	0.68, 0.97	**0.022**
Region			
Urban	–	–	
Rural	1.11	0.95, 1.29	0.18
Baseline alcohol use			
No	–	–	
Yes	1.53	1.15, 2.05	**0.004**
Baseline amphetamine use			
No	–	–	
Yes	1.43	0.39, 5.02	0.57
Baseline antidepressant use			
No	–	–	
Yes	0.67	0.08, 3.72	0.66
Baseline cannabis use			
No	–	–	
Yes	1.18	0.75, 1.82	0.46
Baseline cocaine use			
No	–	–	
Yes	1.34	0.87, 2.03	0.17
Baseline opioid use			
No	–	–	
Yes	1.95	0.68, 5.79	0.21
Baseline other psychoactive substance use			
No	–	–	
Yes	1.7	0.88, 3.22	0.11
Baseline other stimulant use			
No	–	–	
Yes	1.06	0.16, 6.97	0.95
Baseline sedative hypnotic or anxiolytic use			
No	–	–	
Yes	1.56	0.04, 55.6	0.79
Baseline tobacco use			
No	–	–	
Yes	0.89	0.67, 1.18	0.44
Baseline VL			
≤200	–	–	
>200–10000	1.43	1.13, 1.82	**0.003**
>10,000	1.43	1.13, 1.82	**0.003**
Baseline CD4			
<200	–	–	
200–500	1.03	0.89, 1.19	0.74
>500	1.03	0.87, 1.22	0.72
Number of comorbidities			
0	–	–	
≥1	1.5	1.29, 1.73	**< 0.001**

*1 OR* Odds Ratio, *CI* Confidence Interval

Bolded p values are statistically significant at *p* < 0.05

Table 4 shows the associations between sociodemographic and HIV-related characteristics and serious mental health diagnosis. Female sex was associated with approximately two times higher odds of having a serious mental health diagnosis (aOR: 1.87; 95% CI: 1.46–2.41). All minoritized races were associated with lower odds of serious mental health diagnosis compared to White populations, except for others. MSM/IDU populations had higher odds of having a serious mental health diagnosis compared to heterosexual populations. Alcohol use was associated with 78% higher odds of having anxiety compared to individuals not using alcohol (aOR: 1.78; 95% CI: 1.18, 2.66). Having higher viral loads was also associated with higher odds of having a serious mental health diagnosis. Having ≥ 1 comorbidity was also associated with higher odds of a serious mental health diagnosis compared with having no comorbidities. We tested for interactions in the adjusted models and did not find any statistically significant interactions (*p* < 0.05), and therefore no interaction terms were included in the final models.

**Table 4:** Association between sociodemographic, substance use and clinical characteristics and serious mental health diagnosis ever after HIV diagnosis

Characteristic	OR1	95% CI1	*p*-value
Age			
18–24	–	–	
25–39	1.16	0.90, 1.51	0.25
40–59	1.3	0.99, 1.71	0.06
>60	1.26	0.79, 1.96	0.32
Sex			
Male	–	–	
Female	1.87	1.46, 2.41	**< 0.001**
Race/Ethnicity			
White	–	–	
Black	0.55	0.45, 0.68	**< 0.001**
Hispanic	0.18	0.08, 0.34	**< 0.001**
Others	0.63	0.34, 1.08	0.11
Population			
Heterosexual	–	–	
MSM/IDU	1.83	1.27, 2.62	**0.001**
MSM	0.87	0.65, 1.18	0.37
Others	0.82	0.63, 1.07	0.14
Region			
Urban	–	–	
Rural	0.93	0.73, 1.18	0.56
Baseline alcohol use			
No	–	–	
Yes	1.78	1.18, 2.66	**0.006**
Baseline amphetamine use			
No	–	–	
Yes	0.41	0.02, 2.28	0.4
Baseline antidepressant use			
No	–	–	
Yes	0.76	0.04, 5.67	0.81
Baseline cannabis use			
No	–	–	
Yes	1.14	0.60, 2.02	0.68
Baseline cocaine use			
No	–	–	
Yes	1.11	0.62, 1.90	0.7
Baseline opioid use			
No	–	–	
Yes	2.64	0.82, 7.91	0.087
Baseline other psychoactive substance use			
No	–	–	
Yes	1.48	0.63, 3.17	0.34
Baseline other stimulant use			
No	–	–	
Yes	3.3	0.25, 83.8	0.38
Baseline sedative hypnotic or anxiolytic use			
No	–	–	
Yes	6.03	0.19, 191	0.26
Baseline tobacco use			
No	–	–	
Yes	0.81	0.54, 1.20	0.29
Baseline VL			
≤200	–	–	
>200–10000	1.48	1.03, 2.16	**0.036**
>10,000	1.44	1.03, 2.07	**0.041**
Baseline CD4			
<200	–	–	
200–500	1.01	0.81, 1.26	0.91
>500	1.07	0.84, 1.37	0.57
Number of comorbidities			
0	–	–	
≥1	1.41	1.13, 1.74	**0.002**
*1 OR* Odds Ratio, *CI* Confidence Interval			

Bolded p values are statistically significant at *p* < 0.05

## Discussion

This is one of the first studies to examine the associations between sociodemographic and HIV-related characteristics, and mental health diagnoses (common and serious mental health diagnoses) among PLWH in South Carolina. We found that sex, race/ethnicity, population type, alcohol use, and number of comorbidities were associated with having a common mental health diagnosis. In addition, sex, race/ethnicity, alcohol use, viral load and comorbidities were associated with having a serious mental health diagnosis.

Our findings are consistent with previous studies examining factors associated with mental health diagnoses among PLWH. We found that alcohol use was associated with having a common or serious mental health diagnosis. Alcohol use, specifically dependence and heavy use, were found to be associated with higher CES-D scores in crude models [19]. However, after adjusting for age, sex, race/ethnicity (black vs. non-black), homelessness, time since study enrollment, drug use, comorbidities, and other clinical and HIV-related factors, the association between alcohol dependence and depressive symptoms was statistically significant but the association between heavy alcohol use and depressive systems attenuated to non-statistical significance [19].

The current study also did not find a statistically significant association between other substance use and mental health diagnosis. This is in contrast to the findings of DiPrete et al., who found that drug use was associated with poorer depression outcomes along the treatment cascade [20]. Bayes-Marin et al., 2023 found that poor physical quality of life was associated with depression [21] and in the current study, there was a relationship between number of comorbidities and common mental health disorders (depression or anxiety).

Based on a US national sample of PLWH, approximately half reported having a psychiatric disorder, and 40% reported using an illicit drug (other than marijuana) [6]. Bing et al., found that the number of HIV-related symptoms, drug use and dependence, and heavy alcohol use were associated with having a common mental health diagnosis. Our findings are below that found in previous studies where 20% had a mental health diagnosis, 19% had a common mental health diagnosis, 2% reported illicit drug use (other than marijuana). This may imply that mental health conditions may be undiagnosed among the SC population. Our findings also align with Bing et al., where alcohol use was associated with common (and serious) mental health conditions.

There are some limitations that must be considered in the current study. We were unable to examine crucial variables that have been included in previous studies such as social isolation, HIV-related stigma and social support. These variables may also be important factors associated with mental health diagnoses among PLWH in the Southeastern US. Second, these results are from PLWH in South Carolina, and might not be generalizable to PLWH in other geographic regions.

Nevertheless, the study had some strengths. Data on mental health outcomes were based on medical records and clinical diagnoses and were operationalized using ICD-9/10 codes. The operationalization of mental health diagnoses included a wide range of mental health disorders such as schizophrenia, personality disorder, bipolar disorder, schizoaffective disorders, depression, anxiety, obsessive compulsive disorder, dementia, and persistent mood disorder. A wide range of substances were included in the data to determine use of alcohol, amphetamine, antidepressant, cannabis, opioid use, sedative hypnotic or anxiolytic, tobacco, other psychoactive substances and other stimulants. In addition, for the multivariable analyses, we were able to adjust for a wide range of sociodemographic, substance use, and HIV-related characteristics.

## Conclusion

The current study showed that there were varying sociodemographic and HIV-related factors associated with common mental health and serious mental health conditions. Mental health intervention and prevention programs should consider these factors and tailor these programs to specific populations (such as women and MSM/IDU populations) depending on the type of mental health diagnosis. Future research should consider the design and implementation of mental health intervention and prevention programs, especially for PLWH. Future studies can include qualitative and mixed methods approaches to explore and assess potential effect measure modifiers of these relationships, such as moderation by sex and sexual orientation, and how this may impact mental health among PLWH.

## Supplementary Information

Below is the link to the electronic supplementary material.

Supplementary Material 1

## Data Availability

(Data transparency): Data procedures can be obtained by emailing Dr. Jiajia Zhang (jzhang@mailbox.sc.edu).

## Code Availability

(Software application or custom code): Code can be obtained by emailing Jiayang Xiao (xiaoj@email.sc.edu.sc.edu).
