# Supplementary material for: Sociodemographic and HIV-Related Characteristics Associated with Mental Health Diagnoses Among People Living with HIV
Source: AIDS Behav. 2026 Jan 9;30(6):1908–17. doi: 10.1007/s10461-025-05015-z (PMC13303657; doi:10.1007/s10461-025-05015-z)
Supplement: Supplementary file 1 — Supplementary Material 1 [file 10461_2025_5015_MOESM1_ESM.docx]

**Supplemental Table 1. ICD 9 and ICD 10 Codes for Mental Health Disorders**

| **Name** | **ICD9_Code** | **ICD10_Code** |
| --- | --- | --- |
| **Bipolar disorder** | 296.40 |  |
|  |  | F31.0 |
|  |  | F31.10 |
|  | 296.41 | F31.11 |
|  | 296.42 | F31.12 |
|  | 296.43 | F31.13 |
|  | 296.44 | F31.2 |
|  | 296.50 | F31.30 |
|  | 296.51 | F31.31 |
|  | 296.52 | F31.32 |
|  | 296.53 | F31.4 |
|  | 296.54 | F31.5 |
|  | 296.60 | F31.60 |
|  | 296.61 | F31.61 |
|  | 296.62 | F31.62 |
|  | 296.63 | F31.63 |
|  | 296.64 | F31.64 |
|  |  | F31.70 |
|  |  | F31.71 |
|  | 296.7 |  |
|  |  | F31.72 |
|  | 296.45 | F31.73 |
|  | 296.46 | F31.74 |
|  | 296.55 | F31.75 |
|  | 296.56 | F31.76 |
|  | 296.65 | F31.77 |
|  | 296.66 | F31.78 |
|  | 296.89 | F31.89 |
|  |  | F31.81 |
|  | 296.80 | F31.9 |
|  | 296.00 |  |
|  | 296.01 |  |
|  | 296.02 |  |
|  | 296.03 |  |
|  | 296.04 |  |
|  | 296.05 |  |
|  | 296.06 |  |
| **Depression** | 296.20 | F32.9 |
|  | 296.21 | F32.0 |
|  | 296.22 | F32.1 |
|  | 296.23 | F32.2 |
|  | 296.24 | F32.3 |
|  | 296.25 | F32.4 |
|  | 296.26 | F32.5 |
|  | 296.30 | F33.9 |
|  | 296.31 | F33.0 |
|  | 296.32 | F33.1 |
|  | 296.33 | F33.2 |
|  | 296.34 | F33.3 |
|  | 296.35 | F33.41 |
|  | 296.36 | F33.42 |
|  | 311 |  |
| **Schizophrenia** | 295.80 |  |
|  | 295.81 |  |
|  | 295.82 |  |
|  | 295.83 |  |
|  | 295.84 |  |
|  | 295.85 |  |
|  |  | F20.89 |
|  | 295.30 | F20.0 |
|  | 295.10 | F20.1 |
|  | 295.20 | F20.2 |
|  | 295.90 | F20.3 |
|  | 295.60 | F20.5 |
|  |  | F20.9 |
| **Anxiety** |  | F40.00 |
|  | 300.21 | F40.01 |
|  | 300.22 | F40.02 |
|  |  | F40.10 |
|  |  | F40.11 |
|  | 300.23 |  |
|  |  | F40.210 |
|  |  | F40.218 |
|  | 300.29 |  |
|  |  | F40.220 |
|  |  | F40.228 |
|  |  | F40.230 |
|  |  | F40.231 |
|  |  | F40.232 |
|  |  | F40.233 |
|  |  | F40.240 |
|  |  | F40.241 |
|  |  | F40.242 |
|  |  | F40.243 |
|  |  | F40.248 |
|  |  | F40.290 |
|  |  | F40.291 |
|  |  | F40.298 |
|  |  | F40.8 |
|  | 300.20 | F40.9 |
|  | 300.01 | F41.0 |
|  | 300.02 | F41.1 |
|  |  | F41.3 |
|  | 300.09 | F41.8 |
|  | 300.00 | F41.9 |
|  | 309.21 | F93.0 |
| **Dementia** | 294.10 | F02.80 |
|  | 294.11 | F02.81 |
|  |  | F01.50 |
|  |  | F01.51 |
|  | 290.40 |  |
|  | 290.41 |  |
|  | 290.42 |  |
|  | 290.43 |  |
|  |  | F01.0 |
|  |  | F01.1 |
|  |  | F01.2 |
|  |  | F01.3 |
|  |  | F01.8 |
|  |  | F01.9 |
|  |  | F02.0 |
|  | 290.0 |  |
|  | 290.10 |  |
|  | 290.11 |  |
|  | 290.13 |  |
|  | 290.20 |  |
|  | 290.21 |  |
|  | 290.8 |  |
|  | 290.9 |  |
|  |  | F03.90 |
|  | 294.21 | F03.91 |
|  | 331.19 | G31.09 |
|  | 331.82 | G31.83 |
|  | 290.3 | F03.90 |
| **Schizoaffective disorders** | 295.70 | F25.9 |
|  | 295.71 |  |
|  | 295.72 |  |
|  | 295.73 |  |
|  | 295.74 |  |
|  | 295.75 |  |
|  |  | F25.0 |
|  |  | F25.1 |
|  |  | F25.8 |
| **Persistent mood disorder** | 301.13 | F34.0 |
|  | 300.4 | F34.1 |
|  |  | F34.81 |
|  |  | F34.89 |
|  |  | F34.9 |
| **Obsessive compulsive disorder** | 300.3 |  |
|  |  | F42.2 |
|  |  | F42.3 |
|  |  | F42.4 |
|  |  | F42.8 |
|  |  | F42.9 |
| **Personality disorder** | 301.0 | F60.0 |
|  | 301.3 | F60.3 |
|  | 301.4 | F60.5 |
|  | 301.6 | F60.7 |
|  | 301.7 | F60.2 |
|  | 301.9 | F60.9 |
